# Bayesian versus frequentist statistical inference for investigating a one-off cancer cluster reported to a health department

**DOI:** 10.1186/1471-2288-9-30

**Published:** 2009-05-11

**Authors:** Michael D Coory, Rachael A Wills, Adrian G Barnett

**Affiliations:** 1 School of Population Health, Mayne Medical School, University of Queensland, Herston, Australia; 2 Statistical Analysis Unit, Queensland Department of Health, Brisbane, Australia; 3 School of Public Health and Institute of Health and Biomedical Innovation, Queensland University of Technology, Kelvin Grove, Australia

## Abstract

**Background:**

The problem of silent multiple comparisons is one of the most difficult statistical problems faced by scientists. It is a particular problem for investigating a one-off cancer cluster reported to a health department because any one of hundreds, or possibly thousands, of neighbourhoods, schools, or workplaces could have reported a cluster, which could have been for any one of several types of cancer or any one of several time periods.

**Methods:**

This paper contrasts the frequentist approach with a Bayesian approach for dealing with silent multiple comparisons in the context of a one-off cluster reported to a health department. Two published cluster investigations were re-analysed using the Dunn-Sidak method to adjust frequentist *p*-values and confidence intervals for silent multiple comparisons. Bayesian methods were based on the Gamma distribution.

**Results:**

Bayesian analysis with non-informative priors produced results similar to the frequentist analysis, and suggested that both clusters represented a statistical excess. In the frequentist framework, the statistical significance of both clusters was extremely sensitive to the number of silent multiple comparisons, which can only ever be a subjective "guesstimate". The Bayesian approach is also subjective: whether there is an apparent statistical excess depends on the specified prior.

**Conclusion:**

In cluster investigations, the frequentist approach is just as subjective as the Bayesian approach, but the Bayesian approach is less ambitious in that it treats the analysis as a synthesis of data and personal judgements (possibly poor ones), rather than objective reality. Bayesian analysis is (arguably) a useful tool to support complicated decision-making, because it makes the uncertainty associated with silent multiple comparisons explicit.

## Background

Health departments and other agencies are regularly asked by the public to investigate a one-off cluster of cancer cases; or less commonly birth defects or other health problems. [1-3] The concern is usually that an environmental agent in a neighbourhood, school or workplace is responsible, and that if nothing is done there will be more cases of cancer. These concerns are legitimate and it is part of good and empathetic public-health practice to respond to them. [1-3]

All cases of cancer have causes; the key question is whether the cases in a reported cluster are due to a common cause. [4] If a common cause is identified, then actions can be taken to protect or improve the public's health. There are several examples of this, including angiosarcoma of the liver and vinyl chloride, clear cell vaginal cancer in daughters of women who took di-ethyl stilbestriol, and Kaposi sarcoma and HIV. [5] However, there are numerous reports of clusters to health departments each year and 50 years of cluster investigations show that if a common cause is not apparent from environmental or other investigations, then only rarely is a common cause subsequently identified. [1-3,5]

If a common cause cannot be identified, then an additional question the public often wants answered is whether the cluster is explainable as a chance event or whether it truly represents a statistical excess. [6] If there is a statistical excess, then this could be used as a justification for allocating resources to more in-depth and costly investigations or perhaps to a case-control study to assess putative exposures in a group of people with characteristics similar to those from whom the cluster arose.

Assessing whether there is a statistical excess is not straightforward for a variety of reasons; prominent among these is that the boundaries of the cluster in time, space, and person are usually defined after the event (the Texas sharp-shooter problem). [4] This is a particular example of the problem of silent multiple comparisons, which is among the most difficult statistical problems faced by scientists. [7]

Visible multiplicities, such as occur with pre-specified subgroup analyses or sequential monitoring of trials are difficult enough, but at least in these circumstances the researchers (and users of the research) know how many multiple comparisons were under consideration. Much more difficult are silent multiplicities such as occur with publication bias [8] or reporting bias, [9] where users of the research do not know how many multiple comparisons should be considered.

The Texas sharp-shooter problem is an example of silent multiplicity because any one of hundreds, or possibly thousands, of neighbourhoods, schools, or workplaces could have reported the cluster, which could have been for any one of several types of cancer or any one of several time periods. Although many scientists consider it important to adjust for these silent or implied multiple comparisons, the number to adjust for can only be a subjective "guesstimate".

Given these difficulties, some critics of traditional cluster investigations have suggested that assessment of whether the cluster represents a statistical excess is irrelevant and that investigators should concentrate on environmental and other investigations to identify a common cause. [4] Others have argued that the role of health departments (or other agencies) is to resolve the cluster to the satisfaction of the community and that most communities want to know whether the cluster represents a statistical excess. [6,10] Not surprisingly, all the cluster investigation protocols (that we could locate) include a step that assesses the statistical significance of the cluster. [11-14]

The aim of this paper is to contrast the frequentist approach (*p*-values, confidence intervals) with a Bayesian approach (credible intervals) for assessing the role of chance in cluster investigations (i.e., whether the cluster represents a statistical excess). Our main claim is that the Bayesian approach makes the uncertainty associated with silent multiple comparisons explicit and when used with a portfolio of priors is a useful statistical tool to inform complicated decision-making.

## Methods

### Standardized incidence ratio (SIR)

In cluster investigations, it is of interest to know whether the rate associated with the cluster is higher than the rate for the rest of the population. The usual way of reporting such a comparison is to use the standardized incidence ratio:

where, *O *is the observed number of cases reported for the cluster, and *E *is the expected number of cases that would have occurred if the age-specific rates for the entire population (say a state or country) applied to the neighbourhood, workplace or school reporting the cluster. Age-specific rates for an entire population are usually obtained from the relevant population-based cancer registry.

### Case studies

To compare and contrast frequentist versus Bayesian methods, we used observed and expected values from two clusters from Australia. The first is a cluster of leukaemia cases from the Illawarra area (observed = 12, expected = 3.49; SIR = 3.44). [15] The second is a cluster of breast cancer cases at the Australian Broadcasting Corporation (ABC) studios in Brisbane (observed = 10, expected = 1.6; SIR = 6.25). [16] In both situations, these were one-off clusters reported by concerned members of the public to the local health department for investigation.

### Frequentist calculations

To make statistical inferences about the SIR within the frequentist framework we used *p*-values and confidence intervals based on an exact relationship between the chi-squared distribution and the Poisson distribution. [17] Because of the discrete nature of the Poisson distribution, we calculated two-sided *p*-values as twice the probability associated with the upper tail, so that the threshold of *p *= 0.05 corresponded to whether 95% confidence interval included the null value (SIR = 1.0). Common ways to adjust for multiple comparisons within the frequentist framework include the Bonferroni and Dunn-Sidak adjustments. [18,19] We used the Dunn-Sidak adjustment in this paper, that is:

where *n *is the number of multiple comparisons.

Alternatively the confidence interval can be made wider using:

where 1 - *α*_unadjusted _is the percentage coverage for the confidence interval, which is usually specified as 95%.

### Bayesian calculations

We used a method based on the Gamma distribution, which is the conjugate for the Poisson distribution. Conjugate distributions are those where the distributional form of the posterior is the same as that of the prior, but with updated parameters that depend on the data at hand. This is convenient in that it simplifies the calculations, which can be done in a spreadsheet (see Additional file 1).

The Gamma distribution has the form:

If the prior distribution for the SIR is specified as Gamma(*α*, *β*) then, the posterior distribution for the SIR is Gamma(*α *+ *O*, *β *+ *E*). Here, as before, *O *is the observed number of cases reported for the cluster and *E *is the expected number of cases. [14]

When *α *< 1, the Gamma distribution is exponentially shaped and asymptotic to both the vertical and horizontal axes. When *α *= 1, the Gamma distribution is the same as an Exponential distribution. For *α *> 1, the Gamma distribution assumes a uni-modal shape, and for values of *α *less than about 20 it has an obvious skew to the right, as shown by mode < median < mean (Table 1). As *α *increases, the skewness (and variance) decreases and the distribution becomes more symmetric and the mode, median and mean approach the same value.

**Table 1:** Characteristics of selected Gamma distributions with mode = 1.

*α*	*β*	Standard deviation	Mode	Mean	Median	95% prior interval
**1.46**	**0.46**	**2.63**	**1.00**	**3.17**	**2.49**	**(0.22, 10.0)**
2.17	1.17	1.26	1.00	1.85	1.58	(0.25, 5.0)
**2.68**	**1.68**	**0.97**	**1.00**	**1.60**	**1.40**	**(0.29, 4.0)**
3.84	2.84	0.69	1.00	1.35	1.24	(0.36, 3.0)
5.23	4.23	0.54	1.00	1.24	1.16	(0.41, 2.5)
8.64	7.64	0.38	1.00	1.13	1.09	(0.51, 2.0)
12.92	11.92	0.30	1.00	1.08	1.06	(0.58, 1.75)
**24.04**	**23.04**	**0.21**	**1.00**	**1.04**	**1.03**	**(0.67, 1.5)**
77.93	76.93	0.11	1.00	1.01	1.01	(0.80, 1.25)

Mode, Mean, Median and 95% prior interval for the SIR. Priors used in this paper are in bold.

Because the Gamma distribution is skewed for small values of *α *(say < 20), we used the mode, (*α *- 1)/*β*, as the measure of the average SIR, rather than the mean or the median. For consistency, we also used the mode as the measure of the average of the posterior distribution. We specified the prior average (mode) as SIR = 1, which indicates that the cases in the cluster do not have a common cause. Our uncertainty about whether the cases in the cluster have a common cause is reflected in the variance (spread) of the Gamma distribution; smaller variances as reflected by narrower 95% prior intervals (Table 1) mean more certainty that there is not a common cause.

A Gamma(0.001, 0.001) is a standard way of specifying a non-informative Gamma prior. [20] The 95% credible interval obtained after specifying a non-informative prior will be similar to the 95% confidence interval obtained from a frequentist analysis; which is why some statisticians consider that frequentist analysis is a particular type of Bayesian analysis where the prior information is zero. In the context of cluster investigations, a non-informative prior implies that nothing is known about possible values of the SIR, other than the observed and expected values.

However, values of the SIR greater than 10 are unlikely, given that a "strong" association in non-communicable disease epidemiology is typically characterised as one where the exposure increased the risk of disease by about 10-fold (e.g., smoking and lung cancer) [21] and most positive associations are not nearly as strong (e.g., 1.5–4.0).

To explore subjective beliefs about whether the cases in a cancer cluster might be due to common cause (in the absence of an identified exposure) we specified three priors, all with mode = 1.0 and 95% prior intervals of decreasing width (0.22 to 10.0; 0.29 to 4.0; 0.67 to 1.5) reflecting increasing certainty that the cases do not have a common cause. These are given in bold in Table 1.

There are a multitude of reasonable priors that could have been specified. We chose these three because they typify three general situations that might occur if environmental or other investigations did not identify a common cause, but of course other priors could be used. The (0.67, 1.5) prior is the most sceptical of the three (i.e., the most certain that the cases in the cluster do not have a common cause) and could be used in a situation where environmental or other investigations had all but ruled out a common cause. The (0.29, 4.0) prior could be used if environmental and other investigations raised the distinct possibility that there might be a common cause (but as yet had failed to identify one). Finally, the (0.22, 10.0) prior would only occasionally be a sensible choice (given that a common cause had not already been identified by environmental or other investigations) because it is only minimally sceptical and only specifies that SIRs > 10.0 are unlikely. We have included it because it provides a useful reference point and might occasionally be justifiable if there was a very strong suspicion of a common (but as yet unidentified) causal factor that was common to all the cases.

Adjustments, for silent multiple comparisons can be made within a Bayesian framework however, as per the frequentist framework, they require that the number of multiple comparisons is known. [22,23] Empirical-Bayes adjustments for multiple comparisons are also possible, [24] but they require data to calculate SIRs for all of the sub-units that make up the multiple comparisons. This might be feasible if there was a cluster reported from a small geographic area and there were data available for all other similar geographic areas in the state or country (and perhaps other cancer sites and time periods). However, it is not feasible for most clusters, especially those reported from worksites or schools.

Unlike the frequentist approach, the Bayesian approach provides an alternative way of addressing the issue of multiple comparisons. The reasoning is as follows: if we regard the prior as our subjective belief or degree of uncertainty (independent of the observed and expected values) that the cases in a cluster are due to a common cause, then we could regard such a prior as taking into account the silent multiple comparisons.

## Results and Discussion

### Frequentist approach

Table 2 shows the adjusted confidence intervals for the Illawarra and ABC clusters for different numbers of silent multiple comparisons in the frequentist framework. The frequentist approach to silent multiple comparisons is to guess the number of silent comparisons and adjust the confidence interval accordingly. For the Illawarra cluster is it the number of other cities in Australia with a factory that produces benzene? This is about 50 and would mean that the cluster did represent a statistical excess (adjusted confidence interval of 1.16 to 7.71 does not include the null value of 1.0; adjusted *p*-value = 0.028). Or, should it be the number of other local government areas in Australia? This is 647 and would mean the cluster did not represent a statistical excess (adjusted confidence interval: 0.87 to 8.91, adjusted *p*-value = 0.304). A further consideration is whether we should adjust for other types of cancers or other time-periods.

**Table 2:** Frequentist confidence intervals and *p*-values adjusted for silent multiple comparisons, Illawarra and Brisbane ABC cancer clusters

	Illawarra, leukaemia observed = 12, expected = 3.49 SIR = 3.44	Brisbane, ABC, breast cancer observed = 10, expected = 1.6 SIR = 6.25
		
Number of comparisons	Confidence interval (*p*-value)	Confidence interval (*p*-value)
1	1.78, 6.01 (0.00056)	3.00, 11.49 (0.000014)
10	1.30, 7.27 (0.0056)	2.11, 14.10 (0.00014)
100	0.99, 8.40 (0.0548)	1.55, 16.44 (0.0014)
500	0.83, 9.13 (0.2456)	1.27, 17.98 (0.0071)
1000	0.77, 9.44 (0.4309)	1.16, 18.62 (0.0142)
5000	0.65, 10.14 (0.9403)	0.96, 20.08 (0.0689)
10000	0.61, 10.43 (0.9964)	0.89, 20.69 (0.1331)
40000	0.53, 11.0 (>0.9999)	0.76, 21.90 (0.4353)
50000	0.52, 11.1 (>0.9999)	0.74, 22.09 (0.5104)

For the ABC cluster, the final report of the Scientific Investigation Panel adjusted for an estimated 40,000 groups of 150 women based on the size of the Australian female population 15–64 years of age. [16] This is not necessarily wrong, but as for the Illawarra cluster, it highlights the subjective nature of the analysis. Specifically, why not just adjust for the number of groups of 150 women in the city of Brisbane, where the workplace was located? Perhaps we should also adjust for different time periods and different types of cancer?

### Bayesian approach

**Figure 1:**
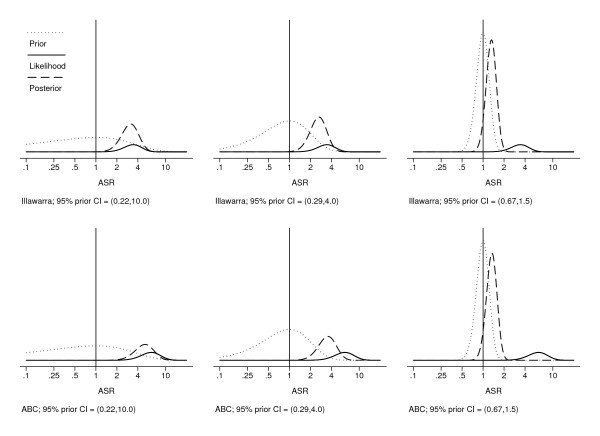
**Posterior distributions for the SIR for three different priors and likelihoods for the Illawarra and ABC clusters**. Dashed lines show the Gamma posterior, solid lines the likelihood, and dotted lines the Gamma prior.

For our case studies, non-informative priors produced results similar to the frequentist analysis, and suggest that both clusters represented a statistical excess (Table 3). More sceptical priors (reflecting more certainty the cases in the cluster do not have a common cause) shrink the posterior mode more towards 1.0 and make it more likely that the 95% credible interval will include the null value of SIR = 1.0 (Figure 1 & Table 3). An appropriate choice for the prior is a matter of judgement and depends on the particular circumstances of the cluster.

**Table 3:** Bayesian analyses for the Illawarra and Brisbane, ABC cancer clusters

	Illawarra, Leukaemia	Brisbane, ABC, Breast cancer
*Uninformative prior*		
posterior mode	3.15	5.62
95% credible interval	(1.78, 5.64)	(3.00, 10.67)
		
95% prior interval (0.22, 10.0)		
posterior mode	3.15	5.08
95% credible interval	(1.84, 5.45)	(2.82, 9.22)
		
95% prior interval (0.29, 4.0)		
posterior mode	2.55	3.56
95% credible interval	(1.53, 4.28)	(2.04, 6.27)
		
95% prior interval (0.67, 1.5)		
posterior mode	1.32	1.36
95% credible interval	(0.95, 1.84)	(0.96, 1.91)

In cases where there is a difference of opinion it is possible to use multiple priors and interpret the posterior intervals in light of the different priors. Sceptical and enthusiastic experts may still disagree about whether the cluster represents a statistical excess, but the different priors hopefully serve to make their prejudice explicit. Using multiple priors can also be a useful sensitivity analysis, and can help to show the influence of the prior. This influence is also shown by the change from the observed SIR to the posterior mean SIR. For the Illawarra cluster concern centred around a local coke by-products plant (which is a major industrial source of benzene); ambient air concentrations of benzene were estimated to have averaged 3 ppb since 1970, or about one-thousandth of the level at which leukaemia risk has been identified in occupational studies. [15] Arguably, a sceptical prior with 95% interval (0.67, 1.5) appropriately captures the tone of the Steering Committee's report, which all but ruled out a common cause. This gives a 95% credible interval of 0.95 to 1.84; which includes the null value of 1.0 and we would conclude that there is no statistical excess. For the ABC cluster, the Scientific Investigation Panel concluded that it was highly unlikely that the cases were caused by exposure, during work on the site, to radio frequency electromagnetic fields, extremely low frequency electromagnetic fields, ionising radiation or chemicals known or suspected to cause breast cancer. [16] However, the Panel was concerned that there might be an unidentified common cause related to the site because the cases were relatively young women who were long-term employees at the site. In the face of this concern, management decided to evacuate the site.

Our assessment of the ABC cluster report is that the Panel would have assigned a larger value to the probability (independent of the observed and expected values) that the cases had a common cause, than the Steering Committee would have for the Illawarra cluster. A reasonable prior might be the (0.29, 4.0) prior, which gives a 95% credible interval of 1.21 to 3.03, suggesting that there is a statistical excess and that further investigations are warranted.

We think there are two main advantages of the Bayesian approach, the most important of which is that it makes uncertainties associated with silent multiple comparisons explicit and incorporates the uncertainty into the statistical analysis. It is easy to see from the sensitivity analysis in Table 3 that conclusions depend on prior beliefs. This explicitly shows that-in the absence of an identified exposure, disagreement among experts is likely, even after thorough and complete investigation of the cluster.

The second advantage of the Bayesian approach is that it allows other uncertainties, such as uncontrolled confounding, to be incorporated into the analysis. For example, the ABC investigation considered other confounders (e.g., alcohol consumption, body mass index, number of children) in a qualitative way, but could not account for them statistically because such data were not available from the population-based cancer registry. [16] If an expert believed that the excess number of breast cancer cases was because of uncontrolled confounding, then a sceptical (0.67, 1.5) prior could be used, giving a 95% credible interval of 0.96 to 1.91. Another, more complicated approach, would be to also put a prior on the expected number of cases, which would require the use of specialised Bayesian software such as WinBUGS. [25]

### Limitations of this study

Some statisticians might claim that a limitation of the approach used in this paper is that we used priors based on the Gamma distribution. Software exists that allows specification of more complicated priors that might be considered more realistic (e.g., WinBUGS [25]). For example, it is possible to use a completely different kind of prior, such as a mixture of two distributions, one with a point-mass in probability at SIR = 1 for the null hypothesis, and another more diffuse prior that captures the alternative. [26] However, we agree with Greenland's argument that such complicated priors are unnecessary for everyday, observational epidemiology, which he accurately describes as semi-quantitative inference about an adjusted risk ratio. [21] In our experience, the computational convenience and simplicity that comes with using the Gamma prior is important for routine work done by health departments.

Another limitation of this study is that we focused on simple data comprising the observed and expected number of cases in a one-off cluster. For richer data with details on multiple locations and spatial information it is advisable to use spatial analyses to put the reported cluster into a geographical context. [26-28]

## Conclusion

The connection between statistics and science has been described as a form of naïve inductive reasoning, [29] which is a view that all scientists seeing the same data would come to the same conclusions. This might be true for a large well-conducted randomised trial, which might eliminate uncertainty and force agreement among experts who might not have agreed before the trial.

Statistical analysis of cluster investigations stray from this ideal to a large, but unknown extent and some experts are sceptical about the existence of a true statistical excess for clusters (in the absence of an identifiable cause). [6] The Bayesian framework allows this sort of prejudice to be displayed explicitly in the prior. On the other hand, if an investigation committee believes that there is a distinct possibility that the cases might have a common cause (perhaps because all the cases are in an unusual age group, as was the case with the ABC cluster), then this can also be displayed explicitly in the prior, which like any aspect of a statistical analysis should be scrutinised and rejected as warranted.

In this way, the subjective Bayesian approach is much less ambitious and less confident than the frequentist approach. The Bayesian approach treats the analysis as a synthesis of data and personal judgements (possibly poor ones), rather than an objective reality. With its portfolio of priors, the Bayesian approach makes uncertainty explicit and is a helpful way of presenting the statistical analysis of a reported cluster.

## Competing interests

The authors declare that they have no competing interests.

## Authors' contributions

MC and RW designed and analysed the study, and wrote the first draft of the manuscript. AB revised the manuscript and critiqued the statistical analysis. RW created the Excel spreadsheet. All authors read and approved the final manuscript.

## Pre-publication history

The pre-publication history for this paper can be accessed here:

http://www.biomedcentral.com/1471-2288/9/30/prepub

## Supplementary Material

Additional file 1 **Bayesian Cluster Analysis Calculator**. Spreadsheet to calculate and plot posterior SIRs given the observed and expected number of cases and the parameters of a Gamma prior distribution. Click here for file

## References

[B1] Caldwell GG Twenty-two years of cancer cluster investigations at the Centers for Disease Control Am J Epidemiol 1990 132 S43 7 2162625 10.1093/oxfordjournals.aje.a115787

[B2] Thun MJ Sinks T Understanding cancer clusters CA Cancer J Clin 2004 54 273 80 10.3322/canjclin.54.5.273 15371285

[B3] Kingsley BS Schmeichel KL Rubin CH An update on cancer cluster activities at the Centers for Disease Control and Prevention Environ Health Perspect 2007 115 165 71 17366838 10.1289/ehp.9021 PMC1797849

[B4] Rothman K A sobering start to the cluster busters' conference Am J Epidemiol 1990 132 1 Suppl S6 S13 2356837 10.1093/oxfordjournals.aje.a115790

[B5] Office of Legislative Policy and Analysis Cancer Clusters, Hearing before the Senate Cancer Coalition. Bethesda 2001 http://olpa.od.nih.gov/hearings/107/session1/reports/cancer_clusters.asp

[B6] Neutra RR Counterpoint from a cluster buster Am J Epidemiol 1990 132 1 8 2356803 10.1093/oxfordjournals.aje.a115621

[B7] Berry D The difficult and ubiquitous problems of multiplicities Pharmaceut Statist 2007 6 155 60 10.1002/pst.303 17879328

[B8] Easterbrook P Berlin J Gopalan R Matthews D Publication bias in clinical research Lancet 1991 337 867 72 10.1016/0140-6736(91)90201-Y 1672966

[B9] Chan A Hrobjartsson A Haahr M Gotzsche P Altman D Empirical evidence for selective reporting of outcomes in randomized trials JAMA 2004 291 2457 65 10.1001/jama.291.20.2457 15161896

[B10] Wartenberg D Why, when and how? J R Statist Soc A 2001 164 13 22 10.1111/1467-985X.00181

[B11] Frumkin H Kantrowitz W Cancer clusters in the workplace: An approach to investigation J Occup Med 1987 29 949 52 3430201

[B12] Leech JA Cancer cluster investigation: toward a more rational approach CMAJ Canadian Medical Association Journal 1989 141 2 105 6 PMC1269331 2743225

[B13] Centres for Disease Control and Prevention Cancer Clusters, Hearing before the Senate Cancer Coalition MMWR 1990 391 1 23

[B14] Fiore BJ Hanrahan LP Anderson HA State health department response to disease cluster reports: a protocol for investigation Am J Epidemiol 1990 132 1 Suppl S14 22 2356826 10.1093/oxfordjournals.aje.a115776

[B15] Westley-Wise V Stewart B Kries I Investigation of a cluster of leukaemia in the Illawarra region of New South Wales, 1989–1996 Med J Aust 1999 171 178 83 10494232 10.5694/j.1326-5377.1999.tb123593.x

[B16] Scientific Investigation Panel ABC. Breast Cancer at the ABC Toowong Queensland http://abc.net.au/corp/pubs/documents/Breast_Cancer_Toowong_Final_Report.pdf

[B17] Ulm K A simple method to calculate the confidence interval of a standardised mortality ratio (SMR) Am J Epidemiol 1990 131 373 5 2296988 10.1093/oxfordjournals.aje.a115507

[B18] Bland J Altman D Multiple significance tests: the Bonferroni method BMJ 1995 310 170 7833759 10.1136/bmj.310.6973.170 PMC2548561

[B19] Leon A Heo M A comparison of multiplicity adjustment strategies for correlated binary endpoints J Biopharmaceut Statist 2005 15 839 55 10.1081/BIP-200067922 16080237

[B20] Congdon P Bayesian Statistical Modelling 2001 New York: John Wiley & Sons, Ltd

[B21] Greenland S Bayesian perspectives for epidemiological research: I. Foundations and basic methods Int J Epidemiol 2006 35 3 765 75 10.1093/ije/dyi312 16446352

[B22] Berry D Hochberg Y Bayesian perspectives on multiple comparisons J Stat Planning and Inference 1999 82 215 27 10.1016/S0378-3758(99)00044-0

[B23] Westfall P Johnson W Utts J A Bayesian perspective on the Bonferroni adjustment Biometrika 1997 84 419 27 10.1093/biomet/84.2.419

[B24] Greenland SRJ Empirical-Bayes adjustments for multiple comparisons are sometimes useful Epidemiology 1991 2 244 51 1912039 10.1097/00001648-199107000-00002

[B25] Lunn D Thomas A Best N Spiegelhalter D WinBUGS – a Bayesian modelling framework: concepts, structure, and extensibility Statistics and Computing 2000 10 325 37 10.1023/A:1008929526011

[B26] Gangnon RE Clayton MK Bayesian Detection and Modeling of Spatial Disease Clustering Biometrics 2000 56 922 935 10.1111/j.0006-341X.2000.00922.x 10985238

[B27] Lawson AB Statistical Methods in Spatial Epidemiology 2006 2 Chichester: John Wiley & Sons

[B28] Pfeiffer DU Robinson TP Stevenson M Stevens KB Rogers DJ Clements ACA Spatial Analysis in Epidemiology 2008 Oxford: Oxford University Press

[B29] Chalmers AF What is this thing called science?: an assessment of the nature and status of science and its methods 1999 3 St Lucia, Qld.: University of Queensland Press

